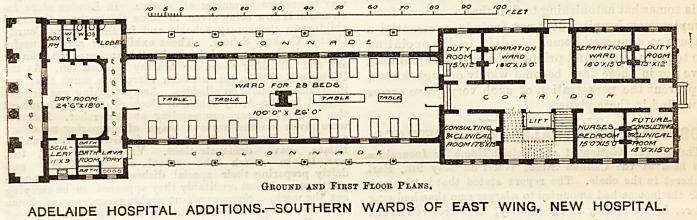# The Adelaide Hospital, Melbourne, South Australia

**Published:** 1895-05-18

**Authors:** 


### The Institutional Workshop.

### HOSPITAL CONSTRUCTION.

### THE ADELAIDE HOSPITAL, ADELAIDE, SOUTH AUSTRALIA.

The plan which we publish to-day represents the first instalment of a scheme for the entire rebuilding of the hospital. The proposed hospital is to consist of three double pavilions, each 300 ft. long, running north and south, and communicating with each other by open colonnades and bridges, and a large separate block for the resident staff. Of this rather more than one half of the three ward pavilions has been completed, and was handed over ready for occupation in March, 1894.

The building consists of the central block of the completed wing, with the southern wards and their adjuncts.

The main wards, of which there are two floors, are each 100 ft. long, 25 ft. wide, and 17 ft. 6 in. high, and contain 28 beds. The beds are arranged at equal distances, with 8 ft. of wall to each bed, but the windows are only between every alternate pair of beds. The cubic space per bed is 1,563 ft, a quite sufficient amount if the floor area were proportionately ample.

cement, painted and varnished, and the ceiling is of finely fluted iron, also painted and varnished. This last is quite a new departure, and has much to recommend it. The difficulty always is to get a ceiling which will not crack, and iron plates seem to promise the very thing needed. We should like to have further details of the mode of jointing the plates and also of the way they are attached. The floors are of kawri pine, oiled. Each ward is warmed by two open fireplaces placed back to back in the centre. The artificial lighting is by gas brackets ranged along the sides of the wards, a plan which has obvious advantages over the more usual method of central pendants.

For ventilation there is a series of grated openings, eight inch by four inch, opening beneath the head of each bed; on each side of the ward six Tobin tubes; and the windows. There are also four large circular-conic openings on each side of the ceiling connected with central shafts passing upwards to the roof, and two large shafts arranged in close proximity to the smoke flues; these are all intended to act as extract ventilators. The committee will, we think, at some not far distant future see ample reason to regret the adoption of Tobin tubes, and will probably about the

Ground and First Floor Plans.

ADELAIDE HOSPITAL ADDITIONS.—SOUTHERN WARDS OF EAST WING, NEW HOSPITAL.

Unfortunately this allowance of cubic space has been obtained by an altogether disproportionate height. In no modern hospital that we can recall has such a height as 17 ft. 6 in. been deemed necessary for the wards; and unless there is some peculiar local condition rendering so unusual a height as this desirable, we cannot but regard it as a most regrettable mistake. The wards, which will be used for clinical teaching would have been more convenient if at least two feet wider, and the addition of two feet linear wall space per bed would have not only given greater separation between the beds, but have allowed the placing of a window between each bed and its neighbours. If, in short, the provision of ample floor area had been first considered, and the question of cubic space made a secondary instead of a primary consideration, the result would have been more satisfactory. It is somewhat strange, too, to find in a modern hospital, and especially one where so much care has been taken to arrive at a perfect plan, that the windows between the corner beds and the end walls of the wards have been forgotten. It is not too late to remedy this defect, which it is to be hoped will not be repeated in the future wards. The walls of the wards are finished with a hard, smooth same time resolve to abolish the eight so-called extract shafts which pass up into the roof.

On each side of the wards are wide balconies with fire escape stairs from the upper wards. At one end of the wards is a long narrow lobby which leads on one hand to the closets, on the other to the bath-room and lavatory, and centrally to a large day-room. The latter opens on to a broad balcony, which must form a most excellent place not only for convalescent patients but for patients in bed whose condition does not preclude their being taken into the open. The day room forms also a passage room to the ward scullery and a box room—an undesirable arrangement which might have been avoided by placing both these rooms in the central block, where they would have been more conveniently situated. The water-closets and sink room are not separated from the wards by an efficiently cross-ventilated lobby, neither do we understand is there any provision for preventing the flow of air towards the ward by keeping up a higher temperature in the closets than in the former—a grave mistake, and one which if not rectified may result in serious disaster. The construction of a ventilated "fæcal cupboard" is the result of a suggestion made by

Professor Allen, of Melbourne University, and is, we may assume, a practical outcome of his recent visit to English hospitals.

The central block contains the staircase, which, on account of the great height of the storey, is unusually large, in the well-hole of which is placed the hydraulic lift for patients. Here also are two separation wards, two nurses' duty rooms, two consultation and clinical rooms, and a bed-room for charge nurses. One of the duty rooms and one of the consultation rooms will be for the service of the northern ward. The floor of this block appears for some reason to have been kept three steps, or some 18 inches, below the floor of the ward. Unless there is some urgent reason connected with the conformation of the ground, such an arrangement will be found to be seriously inconvenient in working, and should, if possible, have been avoided.

We fail to see the meaning of the nurses' duty room, with the ward scullery at the other end of the ward. Both are not required, and the proper place for the ward scullery, or duty room, as it is the fashion to call it, is, as we have before remarked, in the central block, but it should not open directly into the ward.

The position of the charge nurse's bed-room, again, is a mistake; and so also is the placing of a number of nurses' bed-rooms in an upper floor of this block. It is somewhat astonishing to find that a scheme for a complete new hospital does not include so essential a feature as a nurses' home. And it is disappointing too to learn that sound principles of hospital planning have not in these days become widely enough known to prevent the perpetration of such very grave errors as we find in these plans.

## Figures and Tables

**Figure f1:**